# New generation low level laser effect on masseter muscle oxygenation, bite force and algometric changes in myofacial pain syndrome: a randomised, placebo-controlled clinical trial

**DOI:** 10.1007/s10103-024-04272-z

**Published:** 2025-01-27

**Authors:** İrem Karagözoğlu, Nermin Demirkol, Özge Parlar Öz, Sümeyye Yılmaz, Mutlu Özcan

**Affiliations:** 1 Gaziantep University, Gaziantep, Turkey; 2 University of Zurich, Zurich, Switzerland

**Keywords:** Clinical trial, Myofascial pain syndrome, Low level laser therapy, Bite forces, Visual analogue scale, Gaalas laser, Neodymium doped yttrium aluminum garnet lasers

## Abstract

The aim of this study was to compare the effectiveness of different types of low level laser treatment (LLLT) in reducing pain levels, changing oxygen saturation and bite force in patients with myofacial pain syndrome (MPS). 45 patients were randomly assigned to three groups: Group 1 (GRR laser, *n* = 15) received LLLT with Gallium-Aluminium-Arsenide (GaAlAs) diode laser with a wavelength of 904 nm and red laser with a wavelength of 650 nm over masseter muscle region. Group 2 (Nd: YAG laser, *n* = 15) were treated with Neodymium-doped Yttrium Aluminium Garnet laser with a wavelength of 1064 nm and the same protocol with Nd: YAG laser was performed in the Group 3 (placebo, *n* = 15) using sham device. Pain was evaluated by visual analogue scale (VAS), change in oxygen concentration in the masseter muscle was measured by functional near-infrared spectroscopy- fNIRS and bite force was measured with Flexiforce sensors before and after treatment. There was a significant decrease in VAS scores after treatment in all three groups. When pain scores were compared, a greater reduction was seen in the Group 1 and Group 2. The change in oxygen saturation level was not statistically significant in all three groups (*p* > 0.05). Bite force values showed a significant decrease in treatment groups (*p* < 0.05), while there was no significant change in the placebo group (*p* > 0.05). Nd: YAG and GRR laser treatments were effective in reducing the pain caused by MPS and in reducing bite force values. Clinically, GRR laser system provides more effective results with its regional and practical application. ClinicalTrials.gov ID: NCT06442553.

## Introduction

Myofascial pain syndrome (MPS) is a kind of muscular irregularities associated with temporomandibular disorders (TMDs) and also described as regional pain syndrome arising in muscles and muscle fascia, characterized by tenderness to palpation, limited range of motion and the presence of myofascial trigger points (TrPs) [1]. TrPs are hard, hypersensitive points that can be palpated during physical examination. They are located within tight bands of skeletal muscle [2–4]. The main clinical feature of MPS is the presence of sensitive TrPs, painful both at rest and during function. The pain begins when the TrPs are palpated and there is tightness in the muscles [5]. The etiology of MPS is complex and still not fully understood, but a few local and systemic factors have been reported to play a role in the etiology. Occlusal irregularities, temporomandibular joint trauma, parafunctional activities (bruxism), viral infections, vitamin and mineral deficiencies have been reported [6]. Psychosocial factors such as depression and anxiety are also associated with TMD and play an important role in the development of this disease [7].

The mechanism of MPS can be described as: The masticatory muscles, which are constantly active during the day, should rest at night and remove the accumulated lactic acid, but with continuous clenching at night, the lactic acid cannot be removed by the body. The lactic acid accumulated in the masticatory muscles causes pain mediators to be transmitted to the area, the patient feels pain in this area [8, 9]. If diagnosed early, it can be treated with conventional methods, but later it usually requires surgical intervention. There is no single treatment for MPS due to its multifactorial etiology, but the primary goal of local muscle pain treatment is to reduce sensory input to the central nervous system [10].

Several interventions have been proposed to treat MPS, such as pharmacologic therapy including injection of a local anesthetic or saline, botulinum toxin injection, nonsteroidal anti-inflammatory drugs (NSAIDs), antidepressants, benzodiazepine, tramadol, anticonvulsives, and α2-adrenergic agonists, physical therapy including extracorporeal shockwave therapy (ESWT), acupuncture. Non-pharmacologic therapies include needling (dry needling, trigger point injection), occlusal splints, physiotherapy and rehabilitation, ultrasound therapy, transcutaneous electrical nerve stimulation (TENS), relaxation techniques, acupuncture, stretching exercise, mesotherapy, massage therapy, psychological treatment, and low-level laser therapy (LLLT) [11–13].

Low-level laser therapy has analgesic, anti-inflammatory and biostimulatory effects. In principle, the mechanism of action on biological tissue is based on the absorption of visible red and near infrared light by photoreceptors in the cell. Light activates receptors in the cell membrane or mitochondria and converts light energy into chemical energy (ATP), which increases cell function and proliferation [14]. The analgesic and anti-inflammatory effects of the laser are explained by many mechanisms [15]. The biostimulatory effect of the laser can be explained by the polarisation at the cellular level. The permeability of the cell membrane to ions increases with the increase in ATP and cytoplasmic hydrogen at the mitochondrial level [15]. Low-level laser light stimulates the release of many transmitters such as endorphins, nitric oxide, bradykinin, serotonin and prostoglandins, which play a role in pain control. In the case of MPS, the low level laser irradiation, when applied to the entire surface of the masticatory muscles, it increases the microcirculation in this area and thus the blood supply. When the blood supply is increased, the lactic acid can be easily removed by the venous circulation and the lymphatic drainage. As lactic acid can be drained, the brain does not have a stimulus for the transmission of pain mediators and muscle spasms are reduced. The contraction of the muscle is reduced so that the muscle returns to its normal tightness [16].

Lasers are characterized by the wavelength they are in the electromagnetic spectrum [15]. Lasers used in dentistry are grouped as red light with a wavelength of 600–700 nm, near infrared (approximately 700–1000 nm; diode lasers), middle infrared (1200–3000 nm; Er: YAG, Er: Cr: YSGG, Ho: YAG) and far infrared (above 3000 nm; CO_2_ lasers) [15]. Many variations of diode lasers with different wavelengths and properties such as GaAs (Gallium Arsenide), GaAlAs (Gallium Aluminium Arsenide) and Nd: YAG (neodymium doped yttrium aluminum garnet, 1064 nm wavelength) laser are most commonly used for MPS treatment clinically. Nd: YAG laser beam is transmitted via fibre optic cable and produces a point effect in tissue. Therefore, it is necessary to determine the trigger points on muscles for focusing the spot beam. The new generation diode laser (GRR Laser Medikal, RCP01, Ankara, Turkey) enables regional and wide area applications.

There are a number of ways to assess the effectiveness of MPS treatment. Generally, visual analogue scale (VAS), which is a subjective method, has been used in trials. The most common symptom of MPS is pain, but there are other effects as well: muscle hypertrophy, muscle fatigue, ischaemia of muscle tissue, decreased blood flow and damage to muscle fibres [17]. Evaluating the effectiveness of treatment with measurable data, such as occlusal bite force and muscle oxygenation, will provide more precise results. For this reason, both subjective and objective data were compared before and after the treatment in this study.

## Materials and methods

### Study protocol

The study was conducted at Gaziantep University, Faculty of Dentistry, Department of Prosthodontics. The study protocol was approved by the Ethics Committee of Gaziantep University Faculty of Medicine (Decision number: 2022/419) and was performed in line with the principles of the Declaration of Helsinki. The protocol was recorded in National Library of Medicine (ClinicalTrials.gov ID: NCT06442553). A power analysis was performed by G*power (version 3.1.9.2; Germany). An effect size of 0.45, 80% power and a two-sided 5% significance level, the minimum sample size was calculated to be 48 according to the reference study [7]. It was determined that 45 participants would be included in the study (*n* = 15). Each participant signed an informed consent form before the start of the treatment.

### Patient selection and randomization

**Fig. 1:**
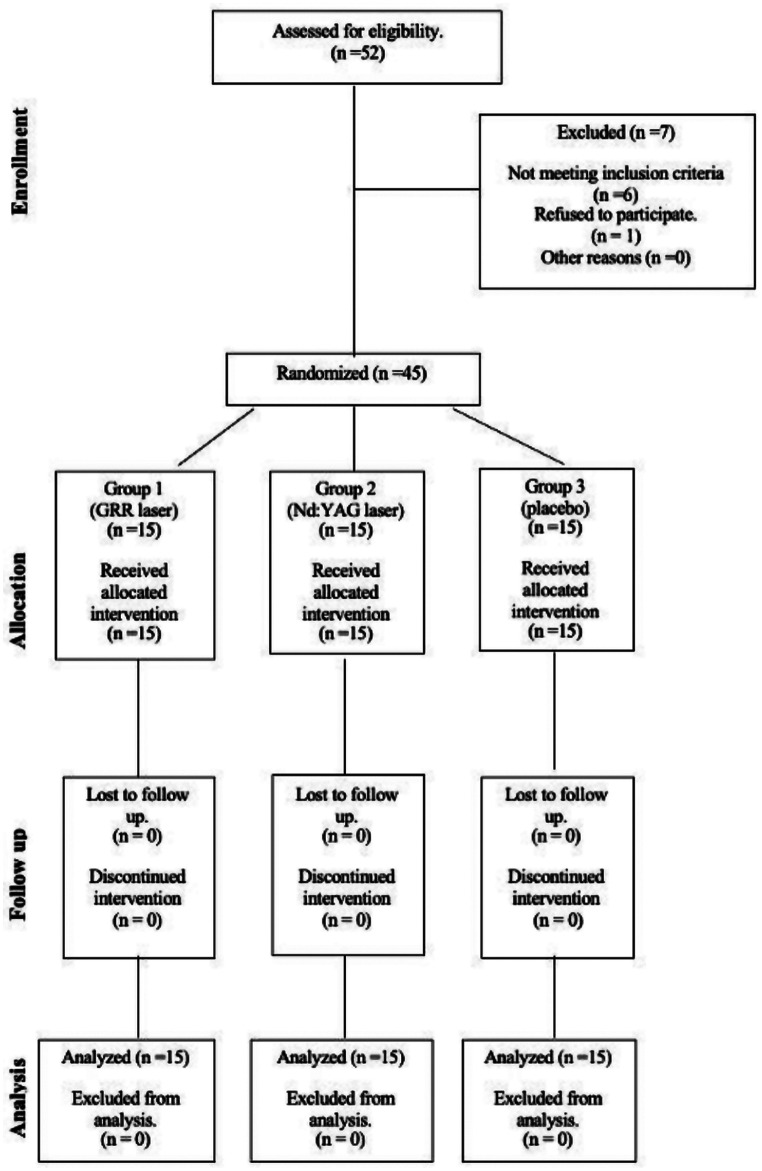
Consort flow diagram

Patients who participated in our study were selected from individuals between the ages of 18 and 25 who presented with symptoms of TMD, were diagnosed with MPS as a result of the clinical examination, and were considered eligible for inclusion in the study. The diagnosis of MPS was made according to the Research Diagnostic Criteria for Temporomandibular Disorders (RDC/TMD) form, which has been tested for reliability and is an international scientific reference [6]. Patients with internal TMJ derangement or degenerative joint changes, patients with restricted mouth opening, deviation or deflection, patients with systemic diseases, pregnant women, patients who had received MPS treatment within the previous year were excluded from the study. A total of 45 patients diagnosed with MPS were randomised into 3 groups. The study was desgined as controlled, prospective, single-blind, randomized study. The randomisation method was carried out using the free research randomiser website developed for researchers. Patients were recruited according to CONSORT (Consolidated Standards of Reporting Trials) criteria. The Consort flow diagram is shown in Fig. 1.

### Laser procedures

**Fig. 2:**
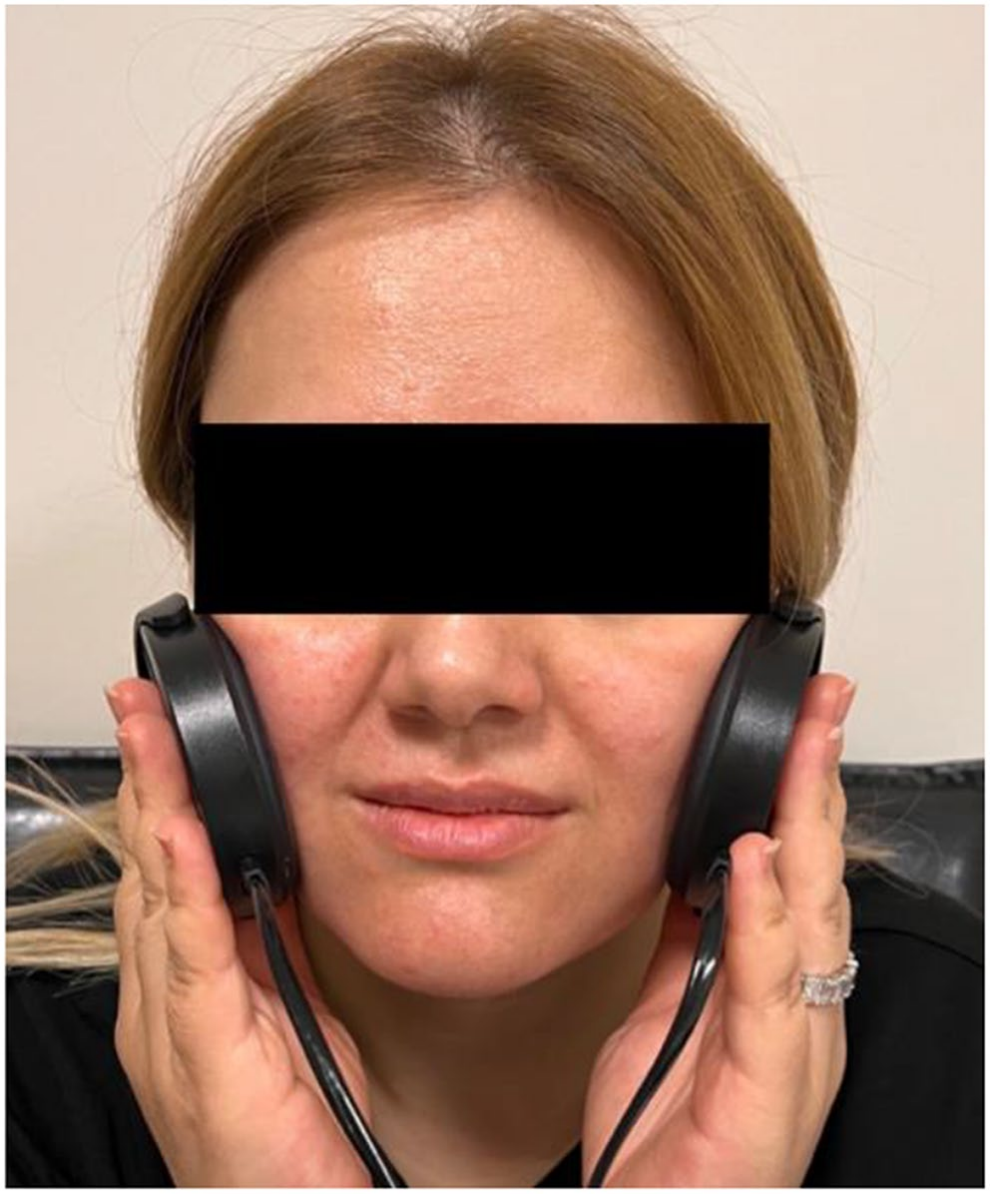
Low-level laser application to the the masseter muscle with the GRR laser headset probe

GRR laser device is a combination of 22 mW output power GaAlAs infrared laser with a wavelength of 904 nm and a 10 mW output power red laser with a wavelength of 650 nm. GRR laser has 4 extraoral probe and one for intraoral application. The diameter of the headset probe is 60 mm and 16 J of energy transfer occurs during one minute application. Treatment procedure was performed in accordance with the manufacturer’s instructions. A total of 15 sessions were applied to each patient for three weeks, five times per week. The headset extraoral probe of the device was placed to cover the entire masseter muscle and applied for 10 min (Fig. 2).

**Fig. 3:**
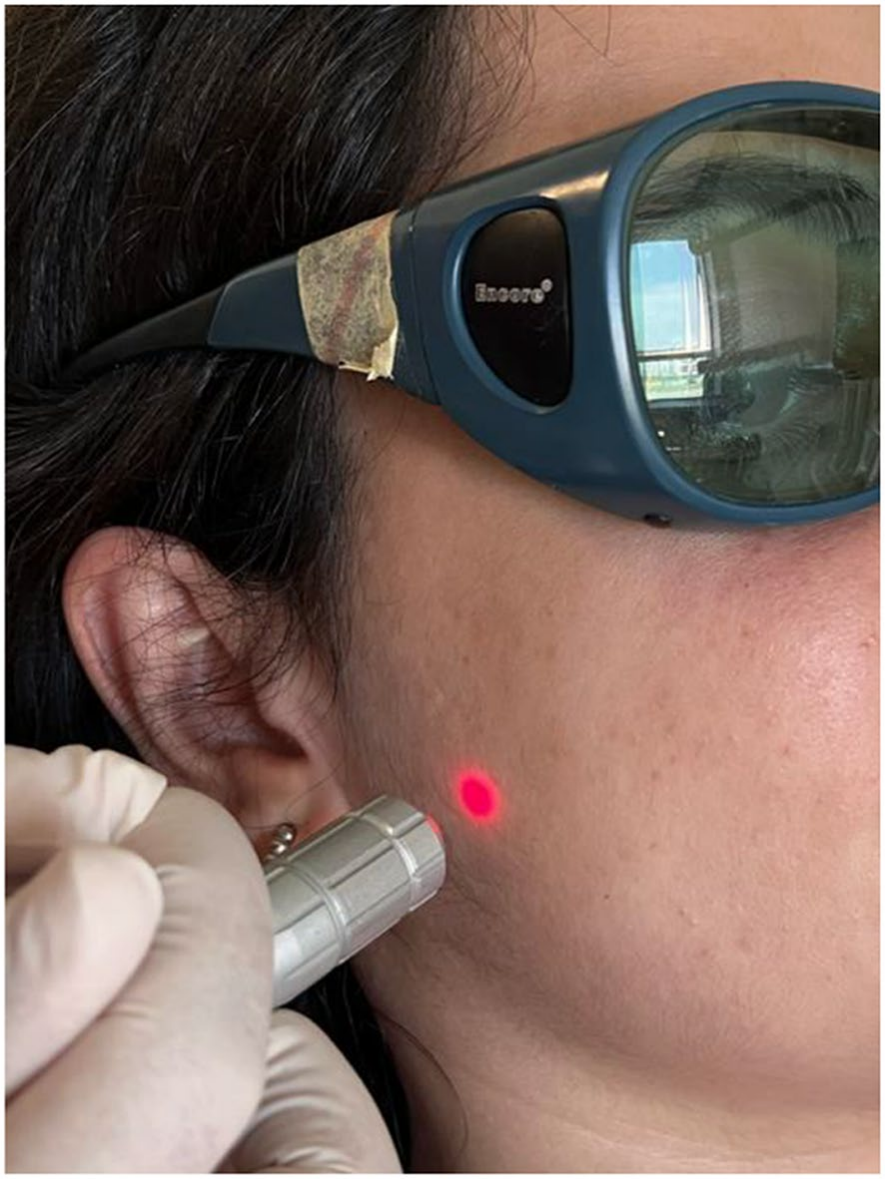
Low-level laser application to the trigger points with the Nd: YAG laser

Nd: YAG laser (Nd: YAG;1064 nm; Fidelis Plus III, Fotona; Ljubljana, Slovenia) procedure; The output power was set to 0.25 W, the duration to 20 s MSP mode with 8 j/cm^2^ energy density. Energy density calculated as: (Watt(W) x second (t)) / laser beam area (cm^2^) The LLLT probe (0.9 cm in diameter) of the device was positioned 1 cm away from the triger points and applied to previously defined three trigger points (Fig. 3). For each patient, a total of 6 points were treated for 2 minutes at each point for 20 s. A total of 10 sessions, five sessions per week, were applied according to reference study [8].

Patients in the placebo group received emission-free laser treatment. In the same procedure with the Nd: YAG laser, a placebo treatment was performed with the device on, laser beams visible but not active.

### Algometric evaluation

Patients included in the study underwent a detailed extraoral palpation to identify trigger points prior to treatment. Palpation was performed on the upper, middle and lower fibres of the masseter muscle with a force of 1 kg for 2 s using two fingers. The areas where the patient felt pain were identified and the intensity of the pain was recorded. Pain was assessed using a visual analogue scale (VAS). Patients were asked to rate the intensity of their pain on a scale from 0 to 10. They were told that a score of 0 meant no pain, a score of 10 meant severe pain and a score of 5 meant moderate pain. Pain scores were recorded by the same clinician immediately before treatment and at the end of treatment for each paient.

### Assessment of muscle oxygenation

**Fig. 4:**
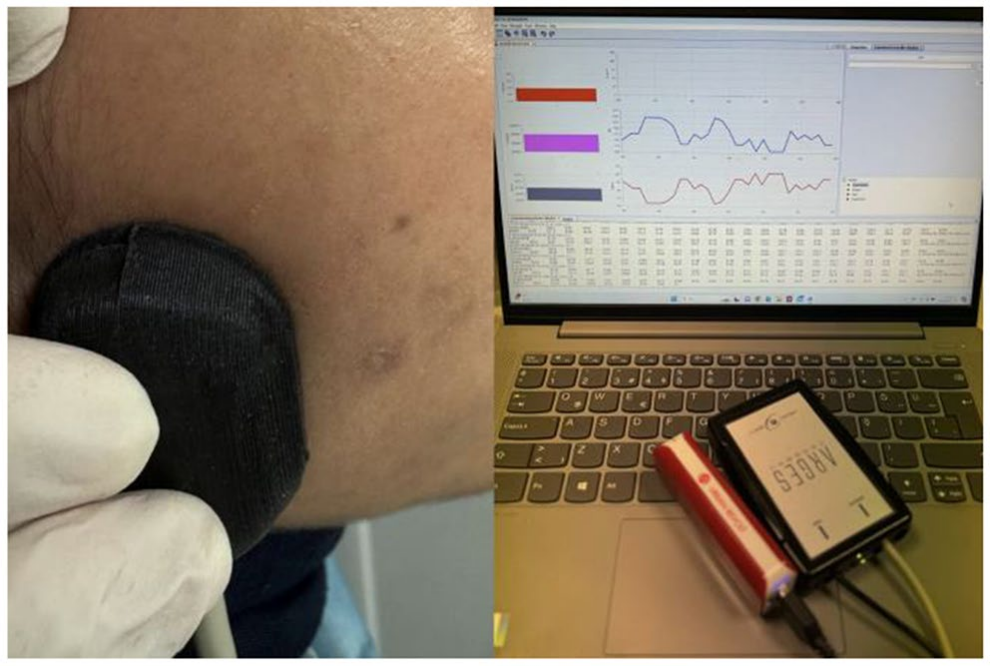
Measurement of masseter muscle oxygenation with functional near-infrared spectroscopy probe

The change in oxygen concentration in the masseter muscle was measured by functional near-infrared spectroscopy- fNIRS (ARGES cerebro, Hemosoft Inc., Ankara, Turkey) before and after treatment for each patient. The NIRS probe consists of near-infrared light emitting diodes (LEDs) and photodetectors. LEDs emit light at two wavelengths (730 and 850 nm). Photodetectors are used to monitor changes in deoxyhaemoglobin (HbO) and oxyhaemoglobin (HbO_2_) concentrations caused by the emitted light. After the ARGES Pro software was installed on the computer, the NIRS probe was placed in the masseter region and a 20 s recording procedure was started. The HbO_2_ value recorded on the computer screen was averaged. The same procedure was repeated at the end of the laser treatments (Fig. 4).

### Assessment of bite force

**Fig. 5:**
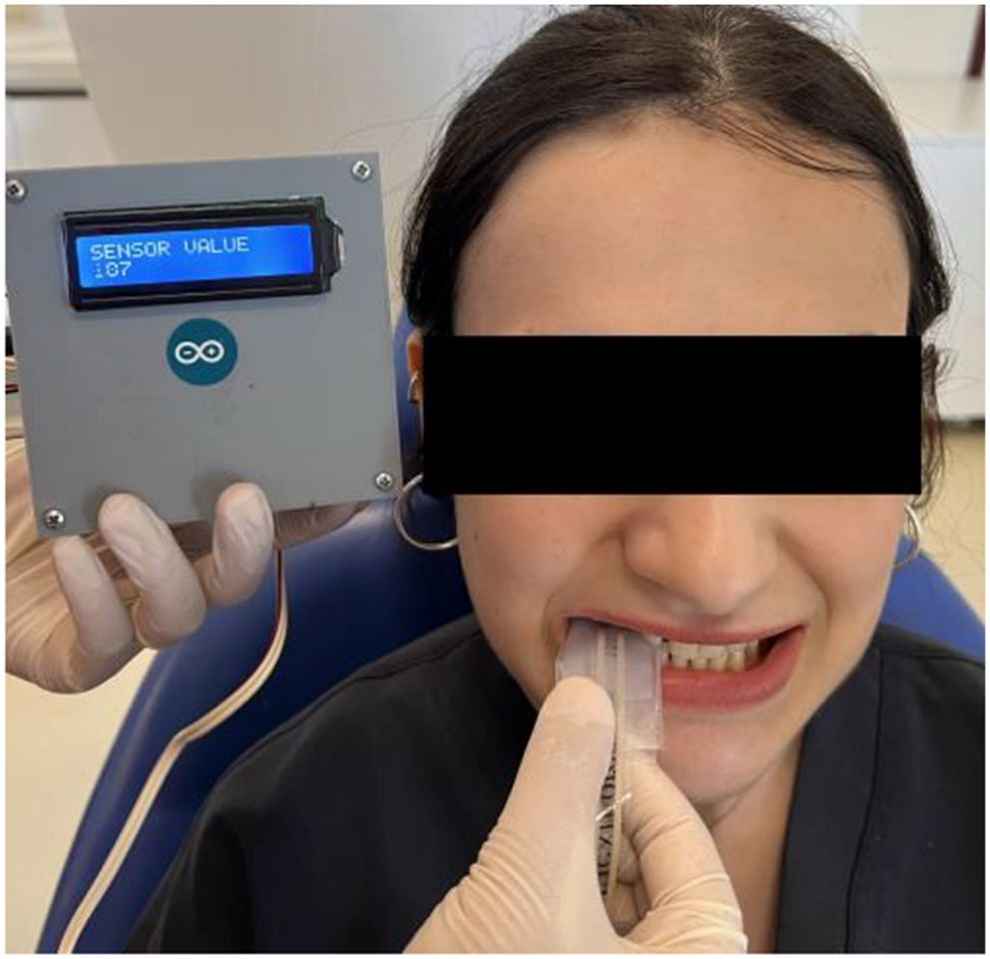
Measurement of bite force with Flexiforce sensors

Flexiforce (Tekscan, South Boston, Massachusetts, United States of America) sensors were placed in a custom-made device. This device has a screen showing the maximum bite force measurement calibrated for Newton (N). The active detection area of the Flexiforce sensors was placed between the first molars with the patients sitting upright in the dentist’s chair and the Frankfurt horizontal plane approximately parallel to the ground (Fig. 5) Patients were asked to bite as hard as they could and the force was recorded for 3–5 s. After allowing the individual to rest for a few seconds, the same measurements were repeated three times and the higher of the three measurements was taken as the maximum bite force for that region. Values were recorded before and after treatment for each patient.

### Statistical analysis

SPSS 29.0 was used to analyse continuous data. Kolmogorov-Smirnov and Shapiro-Wilk tests were used to test whether there was a difference between the distribution of the data and the normal distribution. Paired sample t-test was used to test whether there was a difference between the means of the dependent continuous variables. One-way ANOVA test was used to compare variables that followed a normal distribution between more than two independent groups. Finally, Chi-square test was used to determine whether there was a relationship between two categorical variables. *P* < 0.05 was considered statistically significant.

## Results

This study included 45 patients diagnosed with MPS with a mean age of 21,2 years. Of these patients, 23 were male and 22 were female. No statistically significant difference was found in the demographic data (gender, age), which ensured homogeneity of the data in group formation.

In the GRR laser group, a statistically significant decrease in the VAS variable was found after treatment when comparing the mean values of the variables measured pre- and post-treatment (*p* = 0.001). There was no statistically significant difference between the pre- and post-treatment values for the HbO_2_ variable (*p* = 0.528). There was a statistically significant decrease in the BF variable after treatment (*p* = 0.003) (Table 1).

**Table 1:** Comparison of mean pre- and post-treatment variables in GRR laser group

Paired Samples Statistics^a^							
		Mean	N	Std. Deviation	Std. Error Mean	t	p
Pair 1	VAS1	6,53	15	1,060	,274	10,247	,001
	VAS2	2,53	15	1,125	,291		
Pair 2	HbO_2_1	,025173	15	,0597946	,0154389	,647	,528
	HbO_2_2	,021767	15	,0398279	,0102835		
Pair 3	BF1	425,67	15	114,171	29,479	3,568	,003
	BF2	346,87	15	81,770	21,113		

a. Groups = GRR Laser

When comparing the mean values of the variables measured pre- and post-treatment in the Nd: YAG laser group, a statistically significant decrease in the VAS variable was found after treatment (*p* = 0.001). There was no statistically significant difference between the pre- and post-treatment values for the HbO_2_ variable (*p* = 0.24). A statistically significant decrease was found in the BF variable after treatment (*p* = 0.022) (Table 2).

**Table 2:** Comparison of mean pre- and post-treatment variables in nd: YAG laser group

Paired Samples Statistics^a^							
		Mean	N	Std. Deviation	Std. Error Mean	t	p
Pair 1	VAS1	6,07	15	1,387	,358	7,462	,001
	VAS2	2,67	15	1,633	,422		
Pair 2	HbO_2_1	,031920	15	,0913521	,0235870	0,727	,240
	HbO_2_2	,014120	15	,0194171	,0050135		
Pair 3	BF1	414,93	15	105,216	27,167	2,208	,022
	BF2	344,20	15	107,050	27,640		

a. Groups = Nd: YAG Laser

In the placebo group, a statistically significant difference was found in VAS scores between pre- and post-treatment (*p* = 0.001). There was no statistically significant difference in HbO_2_ and BF scores between pre- and post-treatment (*p* = 0.49, *p* = 0.073) (Table 3).

**Table 3:** Comparison of mean pre- and post-treatment variables in placebo group

Paired Samples Statistics^a^							
		Mean	N	Std. Deviation	Std. Error Mean	t	p
Pair 1	VAS1	7,67	15	1,234	,319	4,298	,001
	VAS2	4,93	15	2,086	,539		
Pair 2	HbO_2_1	,008733	15	,0015486	,0003998	-,026	,490
	HbO_2_2	,008747	15	,0018670	,0004820		
Pair 3	BF1	357,13	15	65,970	17,033	1,536	,073
	BF2	343,80	15	84,667	21,861		

a. Groups = Placebo

**Fig. 6:**
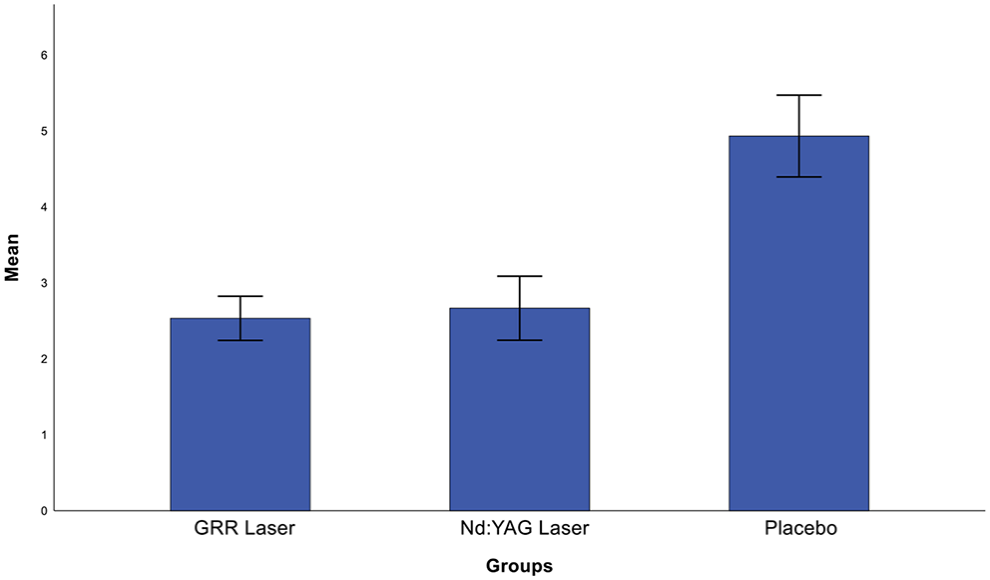
Comparison of post-treatment visual analogue scale scores between groups

**Fig. 7:**
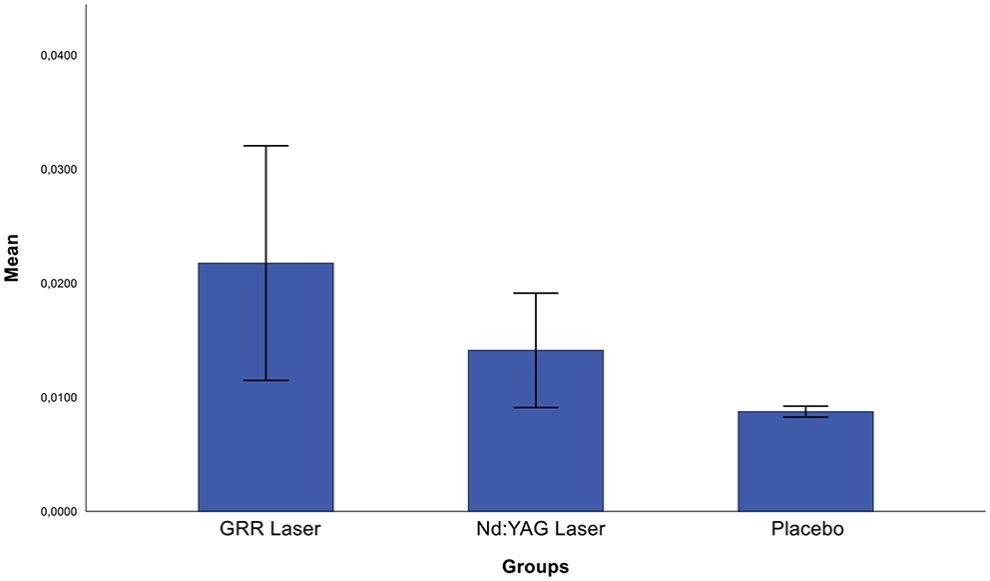
Comparison of post-treatment oxygen concentration scores between groups

**Fig. 8:**
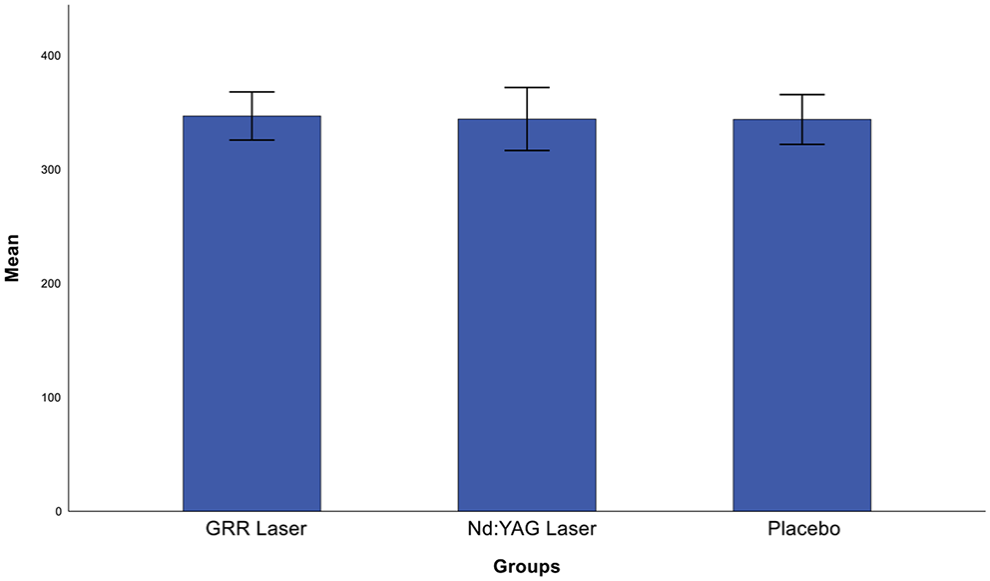
Comparison of post-treatment bite force scores between groups

Post-hoc analysis showed that the mean pain reduction in the GRR laser group was significantly lower than in the placebo group (*p* < 0.05), whereas there was no statistically significant difference with the Nd: YAG laser group (*p* > 0.05). The mean pain reduction in the Nd: YAG laser group was statistically significantly lower than in the placebo group (*p* < 0.05) (Fig. 6). Briefly, there was a significant decrease in VAS scores at the end of treatment, except in the placebo group. When analysing the change in oxygen saturation between the groups after treatment, no statistically significant difference was found between the groups (*p* > 0.05) (Fig. 7). Also, there was no statistically significant difference in bite force values between the groups after the treatments (*p* > 0.05) (Fig. 8).

## Discussion

This study evaluated the effectiveness of two different LLLT devices on patients with MPS using both objective and subjective methods. In order to achieve homogeneity in the study, the patients included in the study were selected from the same age group, social status and profession. Studies show that 55% of patients with maxillofacial pain complain of MPS [18]. Traditionally, temporary treatment with massage, heat, analgesics and myorelaxants has been used to reduce this pain. Treatment options such as intraoral appliances (occlusal splints) or Botox injections are also available [8, 9]. However, these treatments may have some disadvantages. Generally, patients cannot tolerate the use of intraoral appliances, and since Botox application is temporary, it must be repeated every 4–6 months. Since long-term application of Botox paralyses the muscle, the stimulus from the brain is not transmitted to the muscle, muscle tone is affected, and atrophy of the tendons and bone to which the muscle is attached may occur in the future. Therefore, although Botox treatment is effective in the short term, long-term use is not recommended [19]. In recent years, LLLT have also been used to treat TMD [6–8]. Laser equipment has been produced in a variety of models and structures to enable regional application. As the masseter muscle is a large and thick muscle covering the cheek area, there may be a difference in the effectiveness of treatment between regional and point application. Factors such as the laser type, type of application, wavelength and depth of penetration of the laser have a significant effect on the success of the treatment [7]. Therefore, in this study, the efficacy of LLLT was compared using different methods of application, different penetration depths and different wavelengths.

The most common symptom of MPS is pain, but there are other effects as well: muscle hypertrophy, muscle fatigue, ischaemia of muscle tissue, decreased blood flow and damage to muscle fibres [20]. Studies have reported that continuous clenching changes the blood oxygen supply to the masseter muscle [17]. One of the causes of TMD is clenching and grinding of the teeth, if the problem is not solved, the severity of the clenching and grinding will increase [21]. Therefore, the severity of the clenching is expected to decrease after the treatment we apply, and the measurement of the bite force (BF) is a factor that determines the effectiveness of the treatment [21]. Considering all these factors, in the present study, pain level, which is a qualitative method, and bite forces, masseter muscle oxygenation, which allow us to obtain quantitative data, were measured to evaluate the effectiveness of LLLT.

Subjective assessment was performed with a pain scale (VAS) measured by palpation of the masseter muscle. VAS is a proven, reliable measurement method recommended by the American Dental Association (ADA). It has been used in many other studies to measure the pain in the affected area by palpation in joint disorders [22, 23]. In our study, the VAS results showed a decrease in all three groups. However, the reduction in pain level was less in the placebo group than in the treatment groups. As stress is known to be a major factor in the etiology of temporomandibular disorders, we assume that the reduction in pain in the placebo group is due to stress reduction. Because when the other two objective data were analysed, there was no change in the placebo group. The thought of receiving treatment may have caused a reduction in pain levels. In a study by Olavi et al., it was reported that LLLT applied to trigger points significantly reduced the pain level [24]. Similarly, other studies have reported a reduction in pain with LLLT applied to trigger points in MPS, even claiming that the patient can live comfortably for 2–3 years after LLLT [16, 25–28]. However, in the study by Thorsen et al., it was reported that there was no difference in pain level in the treatment group compared to the placebo group [29]. There are many reasons for the differences in these results. There is no definite protocol regarding the applied laser parameters. In the studies, the wavelength, energy density and application time of LLLT are different.

Various lasers with different wavelengths such as 780–904 nm GaAs, 830–904 nm GaAlAs, 1064 nm Nd: YAG have been used in low-level laser studies for TMD [30–34]. The energy density of the laser is also an important parameter. There is no definitive information in the literature on the effective dose for MPS, but 6–10 J/cm^2^ per session have been recommended for myogenic disorders and 4–6 J/cm^2^ per session for arthritis/arthrosis [34]. Studies have generally used an energy density in the range of 3–8 J/cm^2^ at each trigger point [31, 32, 35]. With reference to these studies, we used an energy density of 8 J/cm^2^ for the Nd: YAG laser in our study and successful results were obtained.

In our study, the effectiveness of LLLT was also evaluated with objective data such as bite force and masseter muscle oxygenation. In the literature, the number of studies measuring oxygenation in the masseter muscle is limited and the difference in oxygen level that occurs when the jaws are at rest, clenching or chewing is usually measured [17, 20, 36]. In a study by Puel et al., when healthy individuals and individuals with TMD were compared, it was reported that masseter oxyhaemoglobin values decreased at rest and during contraction, and it is thought that oxygenation decreases with increased contraction of the masseter muscle as a result of stress [37]. In the study by İspirgil et al., haemodynamic changes were observed in the masseter muscle as a result of occlusal splint treatment. It was reported that splint treatment caused a decrease in blood flow and a quantitative decrease in HbO_2_ [38]. In our study, there was no statistical difference in the level of HbO_2_ in both laser treatment groups, but there was a small quantitative decrease, while there was no change in the control group. According to the results of our study, there was a significant decrease in bite force values, which is another quantitative data, in the treatment group. There are no studies in the literature measuring bite force after LLLT, but studies on occlusal splint treatment support the findings of a decrease in EMG amplitude and a decrease in maximum bite force [38].

One of the limitations of the study is the lack of long-term follow-up of the results. Especially in the placebo group, it would have been good to see whether the reduction in pain that occurred immediately after the end of treatment returned to its previous level in the long-term.

## Conclusions

The results of this study showed that both low-level laser treatments, when applied to the painful masseter muscle area, lead to changes in masseter muscle dynamics and are effective in reducing pain levels. Both laser treatments resulted in a reduction in pain level and bite force values, but the reduction of pain level and bite force values were higher with the GRR laser treatment.

## Data Availability

No datasets were generated or analysed during the current study.
